# Genetic Contributors of Efficacy and Adverse Metabolic Effects of Chlorthalidone in African Americans from the Genetics of Hypertension Associated Treatments (GenHAT) Study

**DOI:** 10.3390/genes13071260

**Published:** 2022-07-15

**Authors:** Nicole D. Armstrong, Vinodh Srinivasasainagendra, Lakshmi Manasa S. Chekka, Nam H. K. Nguyen, Noor A. Nahid, Alana C. Jones, Rikki M. Tanner, Bertha A. Hidalgo, Nita A. Limdi, Steven A. Claas, Yan Gong, Caitrin W. McDonough, Rhonda M. Cooper-DeHoff, Julie A. Johnson, Hemant K. Tiwari, Donna K. Arnett, Marguerite R. Irvin

**Affiliations:** 1 Department of Epidemiology, University of Alabama at Birmingham, Birmingham, AL 35294, USA; nmda@uab.edu (N.D.A.); acjones@uab.edu (A.C.J.); rmdeitz@uab.edu (R.M.T.); bhidalgo@uab.edu (B.A.H.); 2 Department of Biostatistics, University of Alabama at Birmingham, Birmingham, AL 35294, USA; vinodh@uab.edu (V.S.); htiwari@uab.edu (H.K.T.); 3 Division of Applied Regulatory Sciences, Center for Drug Evaluation and Research, Silver Spring, MD 20903, USA; lakshmimanasa.chekka@fda.hhs.gov; 4 Department of Pharmacotherapy and Translational Research, College of Pharmacy, University of Florida, Gainesville, FL 32611, USA; namnguyen@ufl.edu (N.H.K.N.); n.nahid@ufl.edu (N.A.N.); gong@cop.ufl.edu (Y.G.); cmcdonough@cop.ufl.edu (C.W.M.); dehoff@cop.ufl.edu (R.M.C.-D.); julie.johnson@ufl.edu (J.A.J.); 5 Medical Scientist Training Program, University of Alabama at Birmingham, Birmingham, AL 35294, USA; 6 Department of Neurology, University of Alabama at Birmingham, Birmingham, AL 35294, USA; nlimdi@uabmc.edu; 7 Department of Epidemiology, College of Public Health, University of Kentucky, Lexington, KY 40506, USA; steven.claas@uky.edu (S.A.C.); donna.arnett@uky.edu (D.K.A.); 8 Division of Cardiovascular Medicine, Department of Medicine, University of Florida, Gainesville, FL 32611, USA; 9 Deans Office, College of Public Health, University of Kentucky, Lexington, KY 40506, USA

**Keywords:** pharmacogenomics, antihypertensives, drug response

## Abstract

Hypertension is a leading risk factor for cardiovascular disease mortality. African Americans (AAs) have the highest prevalence of hypertension in the United States, and to alleviate the burden of hypertension in this population, better control of blood pressure (BP) is needed. Previous studies have shown considerable interpersonal differences in BP response to antihypertensive treatment, suggesting a genetic component. Utilizing data from 4297 AA participants randomized to chlorthalidone from the Genetics of Hypertension Associated Treatments (GenHAT) study, we aimed to identify variants associated with the efficacy of chlorthalidone. An additional aim was to find variants that contributed to changes in fasting glucose (FG) in these individuals. We performed genome-wide association analyses on the change of systolic and diastolic BP (SBP and DBP) over six months and FG levels over 24 months of treatment. We sought replication in the International Consortia of Pharmacogenomics Studies. We identified eight variants statistically associated with BP response and nine variants associated with FG response. One suggestive *LINC02211-CDH9* intergenic variant was marginally replicated with the same direction of effect. Given the impact of hypertension in AAs, this study implies that understanding the genetic background for BP control and glucose changes during chlorthalidone treatment may help prevent adverse cardiovascular events in this population.

## 1. Introduction

Hypertension (HTN) is a leading risk factor contributing to cardiovascular diseases (CVD) worldwide, attributing to ~10.7 million deaths annually [1]. Effective blood pressure (BP) control is critical for the reduction of CVD, as demonstrated in the Systolic Blood Pressure Intervention Trial (SPRINT), where aggressively targeting lower systolic BP (SBP) with antihypertensive treatment (<120 mmHg) versus the standard treatment (<140 mmHg) reduced the risk of myocardial infarction, acute coronary syndromes, stroke, heart failure, or CVD death by 27% [2]. BP control is especially important for African Americans (AA), who have an earlier onset and higher prevalence of HTN compared to individuals of European ancestry (EA) [3,4]. A higher prevalence of HTN is paired with poorer BP control in AAs which cannot be attributed to differences in awareness and treatment or dietary factors [5,6]. 

Previous studies have shown that interindividual and racial differences in BP response to first-line antihypertensive treatments exist [7,8], with ~50% of patients achieving adequate BP response to anyone antihypertensive therapy [9]. Compared to EAs, AA patients have poorer BP lowering response to β-blockers, angiotensin converting enzyme inhibitors (ACEIs), or angiotensin receptor blockers and exhibit a better response to calcium channel blockers (CCB) or thiazide diuretics (TD), when used as monotherapy, thus making CCB or TD the suggested first-line antihypertensive therapy for AAs [7]. While observational studies and randomized trials of BP response have highlighted several metabolic disturbances, hyperglycemia is of great concern [10,11]. In the Antihypertensive and Lipid-Lowering treatment to prevent Heart Attack Trial (ALLHAT), chlorthalidone use increased fasting glucose (FG) by 8.5 mg/dL on average versus an average increase of 5.5 mg/dL in the amlodipine (CCB) group or 3.5 mg/dL for the lisinopril group (ACEI) [12]. This is of importance, particularly in AAs, who have a longer duration of antihypertensive use due to earlier onset HTN, as well as a higher risk of type 2 diabetes (T2D). 

Interindividual BP response to antihypertensives is highly variable at the population level, alluding to a possible genetic component to the antihypertensive response. Genetic studies of BP response to antihypertensive treatment and BP control on antihypertensive treatment are especially vulnerable to medication adherence, as well as noise due to treatment with multiple drugs. Compared to purely observational research of hypertension-related phenotypes, studies set within a clinical trial can minimize many of these limiting factors because participants are randomized to monotherapy first with careful follow-up for BP control and treatment titration as needed using predefined algorithms. Clinical trials also allow evaluation of BP response as a quantitative trait upon new drug exposure (i.e., change in BP from baseline to follow-up), an outcome that is usually not possible to consider using observational data. From a public health standpoint, there is increasing recognition of the need for precision medicine by clinicians, health systems, patients, and policymakers, yet research lags behind this growing demand. Unfortunately, there have been a limited number of pharmacogenetic studies of antihypertensive treatment responses and AAs have been underrepresented in those studies [13]. Candidate gene and genome-wide association studies (GWAS) have uncovered suggestive loci, but a large GWAS of treatment response has not been undertaken in AAs [14,15,16,17,18,19,20,21,22]. Utilizing data from the Genetics of Hypertension Associated Treatments (GenHAT), we aimed to identify genetic variation in the antihypertensive efficacy of chlorthalidone over a six-month period for 4297 AA participants, as well as any genetic risk attributing to changes in FG levels.

## 2. Materials and Methods

### 2.1. Study Population

The GenHAT study is an ancillary pharmacogenomics study to the Antihypertensive and Lipid Lowering Treatment to Prevent Heart Attack Trial (ALLHAT). ALLHAT was a randomized, double-blind, multi-center trial that enrolled 42,418 participants ≥55 years of age with HTN and at least one additional risk factor for CVD [23]. Participants were randomized to treatment with one of four primary antihypertensive drugs: chlorthalidone (thiazide-like diuretic), amlodipine (CCB), lisinopril (ACEI), or doxazosin (α-adrenergic blocker) in a ratio of 1.7:1:1:1, respectively [23]. The original GenHAT study (*n* = 39,114) evaluated the interaction between candidate hypertensive genetic variants and different antihypertensive treatments to modify the risk of fatal and non-fatal CVD outcomes [24]. The current study focuses on 7593 GenHAT participants with genome-wide microarray data. Participants were excluded if they had a genotype missing rate greater than 10% (*n* = 241), if there were discrepancies between self-reported and genotypic sex (*n* = 156), or if they were HapMap controls (*n* = 198), duplicates (*n* = 84), or genetic outliers (*n* = 6) as determined by SMARTPCA [25]. This resulted in a total of 6908 AA GenHAT participants, of which 4297 were randomized to chlorthalidone during ALLHAT. Furthermore, 320 participants were treatment naïve (i.e., they had not been previously treated for their hypertension, T-N) at randomization and were used in sensitivity models. 

### 2.2. ALLHAT Treatment

Randomization drugs were titrated to meet the study treatment goal of SBP less than 140 mmHg and DBP less than 90 mmHg. Chlorthalidone was given once daily at 12.5 mg for the first and second titration and 25 mg for the third, and at the sixth month visit, the average chlorthalidone dose was 16.5 mg [26]. If BP control was not achieved on the maximum dose, a second-step drug (reserpine, clonidine, or atenolol), or a third-step agent (hydralazine) was added at the physician’s discretion [26]. 

### 2.3. Response Phenotypes

The outcomes for the current study were the change in SBP and DBP from baseline to the six-month follow-up visit (ΔSBP = SBP_6 month_ − SBP_Baseline_; ΔDBP = DBP_6 month_ − DBP_Baseline_). Prior to BP measurement, participants were required to sit quietly in an erect, yet comfortable position with their feet flat on the ground for more than five minutes [27]. Blood pressure was measured at each time point and was calculated as the average of two measurements obtained with a 30-s interval between them [28]. Of the 4297 GenHAT participants, 3982 had baseline and six-month BP measures, of which 57% (*n* = 2269) were on chlorthalidone monotherapy at year 1. Of the 320 participants who were treatment-naïve at randomization, 290 had both BP measurements and 66% (*n* = 192) were on chlorthalidone monotherapy at year 1. 

Additionally, we measured the change in fasting glucose (ΔFG) over a 24-month period starting at baseline (ΔFG = FG_24 month_ − FG_Baseline_). Both baseline and follow-up glucose concentrations were obtained after an overnight fast of at least 8 h. Analyses were done at a certified laboratory and detailed previously [12,29]. Of the 4297 GenHAT participants, 1127 had baseline and 24-month fasting glucose measures of which 67% (*n* = 757) were on chlorthalidone monotherapy at year 1 and 54% (*n* = 611) were on chlorthalidone monotherapy at year 2. Due to the small number of treatment naïve participants with two FG measures, secondary analyses on this subset were not performed.

### 2.4. Genotyping and Imputation

Genome-wide genotyping was performed using Illumina Infinium Multi-Ethnic AMR/AFR BeadChip arrays (Illumina Inc, San Diego, CA, USA), comprising over 1.4 million markers. Variants were excluded if they deviated from Hardy-Weinberg equilibrium (*p* < 1 × 10^−5^), had a minor allele frequency (MAF) <0.01, or had a missing rate > 0.01. Furthermore, we analyzed the strand concordance, position, and allele assignment, resulting in 967,857 variants used in imputation. Imputation was performed using the NHLBI Trans-omics for Precision Medicine (TOPMed) Release 2 (Freeze 8) reference panel, which leverages data on >50,000 AA samples [30,31,32]. Variants were excluded with imputation quality (r^2^) < 0.3 or a minor allele count <20, resulting in >20.4 million used in association analysis.

### 2.5. Statistical Analysis

Descriptive statistics are presented as mean ± standard deviation (SD) for continuous traits and counts and percentages for categorical variables and were calculated using SAS version 9.4 (SAS Institute Inc., Cary, NC). Linear regression models were implemented in PLINK2 [33] for genome-wide association analyses of ΔSBP, ΔDBP, and ΔFG, independently, and assumed an additive model of inheritance. The ΔSBP and ΔDBP outcomes were z-transformed, with mean = 0 and SD = 1. The ΔFG outcome was inverse normal transformed using the RNOmni package in R [34]. Models of association between the BP/FG response to chlorthalidone and the imputed effect allele dosage were adjusted for age, sex, top 10 principal components to account for genetic ancestry, and corresponding baseline measure. Regional plots were created using LocusZoom v0.12 [35,36] and gene annotation was performed using ANNOVAR [37]. Genome-wide significance was set at *p* < 5.00 × 10^−8^ after a Bonferroni correction for each response phenotype.

### 2.6. Replication Populations

Replication was sought for both the main and secondary analyses using data facilitated through the International Consortium for Antihypertensive Pharmacogenomics Studies (ICAPS) between 1 February 2021 and 21 May 2021. We utilized genetic and phenotypic data from the Genetic Epidemiology of Responses to Antihypertensives (GERA) study [38], and the Pharmacogenomic Evaluation of Antihypertensive Responses (PEAR and PEAR-2) studies [39,40] (clinicaltrials.gov identifiers NCT00005520, NCT00246519, and NCT01203852, respectively). In PEAR and GERA, participants were randomized to hydrochlorothiazide (HCTZ), while in PEAR-2, participants were randomized to chlorthalidone. All three studies had a washout period of at least 4 weeks to establish hypertensive BPs before starting the drug treatment, making these populations more comparable to our secondary, treatment-naïve population. Furthermore, the response time was shorter for each of these studies in comparison with GenHAT (between 4–9 weeks) [38,39,40]. Details of the individual studies and participant demographics included in replication are available in Supplementary Materials and Table S1.

The number of independent signals, as calculated by linkage disequilibrium (LD) r^2^ > 0.20 per phenotype, was used to calculate a Bonferroni-corrected α level for replication significance. For genome-wide significant discovery variants, replication significance was tested at α = 0.025 divided by the number of significant independent signals. Suggestive variants were tested at α =0.05 divided by the number of independent suggestive signals, as previously described [41].

## 3. Results

The demographics for the GenHAT participants in this study are presented in Table 1. The average age of participants was 66 years and 41% of participants had T2D at baseline. The average baseline SBP and DBP were 146.14 mmHg and 84.86 mmHg, respectively. The average baseline FG was slightly elevated at 127.40 mg/dL and the serum potassium levels were 4.22 mmol/L (Table 1). The 290 participants who were treatment-naïve at baseline were similar in age to the larger population, but 28% had T2D and the average FG levels were lower at 118.59 mg/dL. The average SBP and DBP for these individuals were 155.92 mmHg and 90.43 mmHg, respectively (Table S2).

GenHAT participants saw a reduction of nearly 6.7 mmHg for SBP and 3.1 mmHg for DBP over the six months. For FG there was an average increase of 6.7 mg/dL over 24 months (Table 2). For the subset of treatment naïve participants, larger responses were observed (−19.5 mmHg for SBP, −9.4 mmHg for DBP, and +10.3 mg/dL for FG, Table S3).

**Figure 1:**
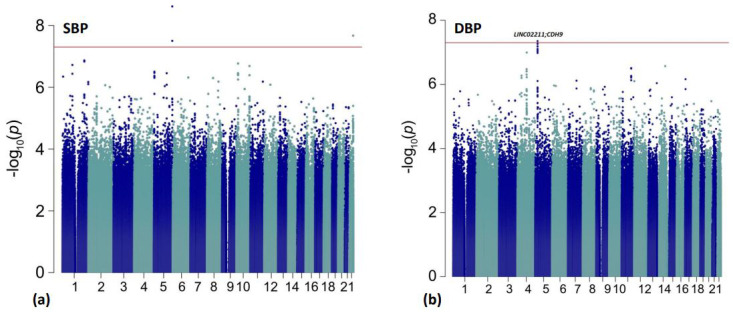
Manhattan plots for the primary discovery analyses for BP response. The red line is representative of genome-wide significance (*p* < 5.00 × 10^−8^). Results are shown from (**a**) ΔSBP analysis; (**b**) ΔDBP analysis.

**Figure 2:**
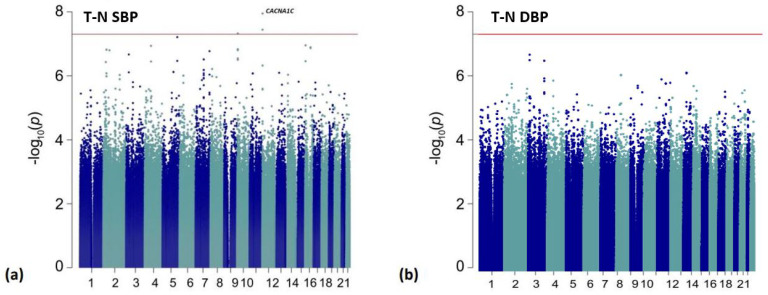
Manhattan plots for the treatment-naïve (T-N) analyses. The red line is representative of genome-wide significance (*p* < 5.00 × 10^−8^). Results are shown from: (**a**) ΔSBP_T-N_ analysis; (**b**) ΔDBP_T-N_ analysis.

Manhattan plots for the primary discovery analysis for BP response (ΔSBP and ΔDBP) are presented in Figure 1. The treatment naïve analysis results (ΔSBP_T-N_ and ΔDBP_T-N_) are reported in Figure 2, and ΔFG is presented in Figure S1. The genomic inflation factor (λ) from the individual analyses ranged from (λ = 0.985 − 1.012) and showed no evidence of systematic inflation (Table S4; Figures S1 and S2).

In Table 3, we present 14 top variants (*p* < 1.00 × 10^−7^) associated with chlorthalidone BP response, 4 of which met the statistical significance criteria after Bonferroni correction (*p* < 5.00 × 10^−8^). An additional 83 variants (42 for ΔDBP and 41 for ΔSBP) were suggestively associated (*p* < 1.00 × 10^−6^) with BP response to chlorthalidone over six months (Tables S5 and S6). The most significant association in the current study was between the rare intronic cadherin related family member 2 (*CDHR2*) variant rs191702725 on chromosome 5 and ΔSBP (*p* = 2.40 × 10^−9^). On average, the GG genotype carriers observed a reduction of 6.79 mmHg over 6 months compared to an increase in BP (or non-response) with the carriers of the A effect allele (AA/GA) (Figure S3A). This rarer variant was not observed in any of the ICAPs data for replication (Table S5). The top variant for the ΔDBP analysis was rs10440665, an intergenic variant between *LINC02211* and *CDH9* on chromosome 5 (G allele, EAF = 26.7%, *p* = 4.48 × 10^−8^; Table 3). At this variant, persons homozygous for the effect allele G observed a 4.19 mmHg reduction in blood pressure compared to a 2.52 mmHg reduction observed in those homozygous for the A allele (*p* = 0.007) (Table 3, Figure S3B). An additional 12 variants in this intergenic region were associated with a stronger DBP response for the effect allele (Table S6; Figure S4). Additionally, two intergenic variants located downstream of *FZD1* were marginally associated with DBP response, rs7802445 (β = −0.84, *p* = 7.67 × 10^−7^) and rs79111780 (β = −0.84; *p* = 7.67 × 10^−7^) and had an EAF ~5%. In consideration of 12 independent signals for DBP response, no variant was replicated (α = 0.05/12). We did observe nominal replication for one of the *LINC02211-CDH9* intergenic variants in the GERA population, rs13164498 (*p* = 4.18 × 10^−2^), with the same direction of effect (Table S6). We also observed marginal replication (*p* < 0.05) in PEAR-2 for both *FZD1* variants (rs7802445 [β = −3.71, *p* = 1.86 × 10^−2^] and rs79111780 [β = −3.67; *p* = 1.97 × 10^−2^]) (Table S6).

In a subset of 290 participants who prior to ALLHAT randomization were not treated with an antihypertensive, we identified four statistically significant variants associated with SBP response (Table 4; Figure 2). Of these, three variants were located within an intron of the CACNA1C gene, with an additional CACNA1C intronic variant reaching suggestive significance (Figure S5). The top variant was rs114758661 and was significantly associated (*p* = 1.12 × 10^−8^) with SBP response. Compared to an increase/non-response in BP with G-allele carriers (GG/GA), non-carriers (AA) had an average reduction of 20.39 mmHg SBP (*p* < 0.0001) (Figure S3C). We did not observe any replication for the CACNA1C variants in the PEAR or PEAR-2 populations (replication threshold for independent significant signals = 0.025/2 independent signals = 1.25 × 10^−2^). Furthermore, we report 19 suggestive intronic variants of LTBP1 in the treatment naïve SBP response analysis (*p*-value ranges from *p* = 1.52 × 10^−7^ to *p* = 8.30 × 10^−7^). There was marginal replication in PEAR for four of these variants (*p* < 0.05) with the same direction of effect (Table S7). Additionally, we observed four variants with a marginal association with ΔDBP in the intergenic region between CYSLTR2 and FNDC3A (*p* = 7.78 × 10^−7^) for the treatment-naïve participants (Table S8). 

In our ΔFG analysis, we observed nine statistically significant variants and an additional 49 variants with *p* < 1.00 × 10^−6^ (Table S9; Figure S1). The top five associated variants were all located within 16 kb of each other in the GIMAP1-GIMAP5 read-through region on chromosome 7 (Figure S6) and have an allele frequency of 0.9%. Of these GIMAP variants, none were replicated with genome-wide significance (α =0.025/5 independent signals for ΔFG =5.00 × 10^−3^) (Table S9).

Additionally, we aimed to replicate previously published variants associated with BP or FG response to thiazide diuretic mono- or combination-therapy in AA or EA populations (Supplementary Tables S10 and S11). We identified 75 variants, mapping to or near 63 unique genes, in the literature dating back to 2010 associated with BP response to thiazide diuretics. We found marginal associations (*p* < 0.05) with three published variants, rs3758785, rs12505746, and rs321320, in the ΔSBP results (*SNN-TXNDC11*, *TET2*, and *CLIC5-RUNX2* regions, respectively); six variants, rs1802409, rs12505746, rs4551053, rs238, rs822127, and rs9590353 in the ΔDBP results (*SNN-TXNDC11, TET2, EBF1, LINC00092, PLCXD3-OXTC1,* and *UGGT2* regions, respectively); and three variants, rs1458038, rs12505746, and rs4757718, in the treatment-naïve ΔDBP results (*FGF5, TET2,* and *PTPN5*, respectively) (Table S10). We identified 40 variants in the literature dating back to 2010 associated with FG response to thiazide diuretics and one with the nominal association in our FG analysis, rs7077606 near *PCDH15* (Table S11).

## 4. Discussion

Antihypertensives are one of the most prescribed medications in the United States, and chlorthalidone is a recommended first-line therapy for treating HTN, particularly in AAs [7]. The discovery of genetic variants that associate with the BP response to chlorthalidone may help improve treatment outcomes. In the current study, we aimed to identify any genetic contributors to the efficacy of chlorthalidone in 4297 AAs from GenHAT. Furthermore, we elucidated genetic variation associated with changes in FG in these participants. Using a genome-wide approach, we identified eight significant variants associated with BP response (3 for ΔSBP, 1 for ΔDBP, and 4 for ΔSBP_T-N_) and nine significant variants associated with FG response to chlorthalidone.

For DBP response, the most significant region was located upstream of *CDH9*. *CDH9* encodes cadherin 9, a type II classical cadherin from the cadherin superfamily, whose function is to mediate cell-cell adhesion [42]. An intronic variant of *CDH9* was previously implicated in coronary artery calcification (CAC) in AAs [43]. Compared to other antihypertensives (CCB nifedipine), thiazide diuretics are associated with a faster progression of CAC in hypertensive patients [44]. Furthermore, *CDH9* has been described as a reliable cell surface marker for renal fibroblasts, the primary matrix-producing cells in the kidney [45,46], which are involved in BP regulation [47]. These previous reports add biological support to this intergenic locus possibly through a regulatory role (given the location of these variants). 

In our treatment-naive secondary analyses, we reported three significant variants in *CACNA1C* for ΔSBP. *CACNA1C* encodes the calcium voltage-gated channel subunit α 1C. Calcium channels mediate the influx of calcium ions into the cell upon membrane polarization [42] and are involved in several physiological processes including vascular and smooth muscle contraction, which is important in BP regulation [48,49]. Genetic variation within *CACNA1C* has been reported to affect CCB efficacy in previous pharmacogenomic studies of HTN [50,51,52]. Findings in over 1700 Han Chinese individuals from the Genetic Epidemiology Network of Salt Sensitivity (GenSalt), reported *CACNA1C* as a potential genetic contributor to BP variation over time in both single-variant and gene-based analyses [53]. 

The variants of additional interest due to their biological plausibility included two intergenic variants downstream of *FZD1* (ΔDBP)*,* 18 intronic variants of *LTBP1* (ΔSBP_T-N_), and four intergenic variants upstream of *CYSLTR2* (ΔDBP_T-N_). The *FZD1* gene encodes the frizzled class receptor 1, a member of the “frizzled” family of membrane domain proteins that functions as receptors for Wnt signaling proteins [42]. The relationship between Wnt/β-catenin signaling and hypertension is well documented, evidenced through genome-wide association studies in AA [14] and EA [54] populations, murine in vivo models [55], and through cardiac function [56,57]. Furthermore, *FZD1* has been independently described as an essential mediator of cardiac hypertrophy (Fan et al., 2018). *LTBP1* encodes the latent transforming growth factor β binding protein 1, a key regulator of transforming growth factor β activation [42]. Anomalous expression of *LTBP1* has been previously detected in thoracic aortic aneurysms [58] and may promote the development of arterial diseases [59]. *LTBP1* has been implicated as a primary regulator of the effect of lethal (3) malignant brain tumor-like protein 4 (L3MBTL4 protein) on vascular remodeling and HTN [60]. The four variants located within an intergenic region of *CYSLTR2* and *FNDC3A* were marginally associated with ΔDBP in treatment-naive participants and were in strong LD (r^2^ > 0.9). *CYSLTR2* encodes the cysteinyl leukotriene receptor 2 (CysLT_2_R). *CYSLTR2* is highly expressed in the heart and coronary vessels [61], and in addition to the cysteinyl leukotriene receptor 1, mediates the actions of inflammatory mediators (e.g., cysteinyl leukotrienes, leukotriene C_4_, leukotriene D_4_, and leukotriene E_4_) affecting smooth muscle construction and vascular permeability [62,63]. A 2004 study performed in endothelial cell specific human CysLT_2_R transgenic mice reported distinct vascular effects of cysteinyl leukotrienes mediated via CysLT_2_R [61].

In the ΔFG analysis, we identified five statistically significant variants in the *GIMAP1-GIMAP5* naturally occurring read-through region. The GIMAPs (GTPases of the immunity-associated proteins) are a family of GTPases that are expressed in the immune system [42]. *GIMAP* GTPase family genes, *GIMAP4* and *GIMAP5*, have been previously described as potential modifiers of immune-mediated diseases, such as type 1 diabetes, asthma, and allergic sensitization [64]. The *GIMAP1-GIMAP5* region was previously associated with CAC in AAs with T2D [65]. To our knowledge, no other study has described genetic variation in this read-through region in relation to the change in FG or BP during antihypertensive treatment. This gene region was not replicated; therefore, additional studies are needed to determine if this locus is relevant to FG changes during treatment.

In the lookup analysis of previously published pharmacogenomic studies on thiazide diuretics and BP, we observed marginal associations with variants located within or near *SNN-TXNDC11*, *TET2*, *CLIC5-RUNX2*, *EBF1*, *PLCXD3-OXTC1*, *UGGT2*, *FGF5*, and *PTPN5*. Of these suggestive associations, the most biologically relevant include variants within *FGF5* (Fibroblast Growth Factor 5), *TET2* (Tet Methylcytosine Dioxygenase 2), *CLIC5* (chloride intracellular channel 5), and the *EBF1* (early B-cell factor 1). *FGF5* has previously been implicated as a susceptibly region for primary hypertension [66,67], while *TET2* has been implicated as an aldosterone responsive mediator of α epithelial sodium channel transcription (which has a role in BP regulation) [68]. *CLIC5* functions in the maintenance of renal glomerular architecture of podocytes and endothelial cells, but it is unknown whether it functions in thiazide action [69,70]. *EBF1* is a transcription factor with the hematopoietic function that has been associated with early onset coronary artery disease [71]. In a large meta-analysis of BP traits, rs4551053, an upstream variant of *EBF1,* was significantly associated with SBP (*p* = 1.17 × 10^−47^), DBP (*p* = 2.98 × 10^−40^), and pulse pressure (2.82 × 10^−23^) in 700,000 EAs [72].

The current study has several strengths. As an ancillary study to ALLHAT, one of the largest antihypertensive clinical trials to date, GenHAT is a rich data source for pharmacogenomic analysis. Additionally, we utilized the ancestrally diverse Illumina Multi-Ethnic array and TOPMed reference panel for imputation which includes ~25% individuals of African ancestry, allowing for improved coverage of African ancestral genomes and rare variant inclusion not possible with previous arrays and a 1000 G reference panel [32]. This study has some limitations as well. As we have noted, we completed secondary analyses on 290 AA participants who were treatment-naive at ALLHAT randomization. While this number is on par with several other published pharmacogenomic studies evaluating thiazide diuretic efficacy [73,74,75], the primary analyses were performed on a population where the majority were already taking an antihypertensive agent. Furthermore, BP measurement is prone to error, but BP for this study was measured twice after a participant had been seated quietly for at least five minutes by trained staff using standardized procedures across all centers in the ALLHAT study. Most of the significant variants we identified had EAF between 1–5% (apart from the intergenic *LINC02211-CDH9* variants), making it difficult to replicate, especially in cohorts with limited genetic data in AAs. Lastly, a common limitation of GWAS are the small effect sizes for the observed results, thus suggesting further validation.

Given the significant burden of HTN in AAs, strategies to improve BP control are of high priority. Identifying genetic variation associated with BP response and/or adverse metabolic effects may lead to drug optimization in these individuals. Although we identified eight variants statistically associated with BP response to chlorthalidone and an additional nine variants associated with the change in FG, none of these results were replicated across all three replication cohorts. Regardless, our findings still have biological plausibility, and further investigation into these variants and gene regions are warranted. Ultimately, understanding the genetic risk factors for both BP control and glucose dysregulation during chlorthalidone treatment continues to be an important research avenue to prevent excess CVD risk in AAs.

## Figures and Tables

**Table 1:** Baseline GenHAT chlorthalidone participant demographics ^1^.

N	4297
Sex, % Female	55.71% (2394)
Age, years	66.14 ± 7.73
BMI, kg/m^2^	30.46 ± 6.54
T2D status	40.66% (1747)
Cigarette smoking status	27.44% (1000)
eGFR, mL/min/1.73 m^2^	82.66 ± 21.49
SBP, mmHg	146.14 ± 15.67
DBP, mmHg	84.86 ± 21.49
FG, mg/dL	127.40 ± 65.30
K, mmol/L	4.22 ± 0.54

^1^ Abbreviations: BMI—body mass index; T2D—type 2 diabetes mellitus; eGFR—estimated glomerular filtration rate; SBP—systolic blood pressure; DBP—diastolic blood pressure; FG—fasting glucose; K—serum potassium.

**Table 2:** Chlorthalidone blood pressure and glucose response ^1^.

	N	Mean Difference ± SD
ΔSBP, mmHg	3982	−6.69 ± 19.30
ΔDBP, mmHg	3982	−3.06 ± 10.82
ΔFG, mg/dL	1127	6.71 ± 58.65

^1^ Abbreviations: SD—standard deviation; SBP—systolic blood pressure; DBP—diastolic blood pressure; FG—fasting glucose.

**Table 3:** Top variants associated with blood pressure response to chlorthalidone use over six months (*p* < 1.00 × 10^−7^) ^1^.

rsID	CHR:BP	A1/A2	EAF	β ^2^	95% CI	*p* ^3^	Location	Gene
ΔSBP								
**rs191702725**	**5:176580424**	**A/G**	**0.003**	**1.08**	**0.73, 1.43**	**2.40 × 10^−9^**	**intronic**	***CDHR2***
**rs6009272**	**22:49475763**	**G/C**	**0.444**	**0.11**	**0.07, 0.15**	**2.14 × 10^−8^**	**intergenic**	***LINC01310; MIR3667***
**rs186198403**	**5:176649236**	**A/G**	**0.003**	**1.02**	**0.66, 1.39**	**3.15 × 10^−8^**	**intronic**	***TSPAN17***
**ΔDBP**								
**rs10440665**	**5:25918567**	**G/A**	**0.267**	**−0.11**	**−0.15, −0.07**	**4.48 × 10^−8^**	**intergenic**	***LINC02211; CDH9***
rs1593983	5:25919837	A/G	0.267	−0.11	−0.15, −0.07	5.18 × 10^−8^	intergenic	*LINC02211; CDH9*
rs28413118	5:25929421	A/C	0.270	−0.11	−0.15, −0.07	5.37 × 10^−8^	intergenic	*LINC02211; CDH9*
rs10050387	5:25928779	C/T	0.268	−0.11	−0.15, −0.07	5.44 × 10^−8^	intergenic	*LINC02211; CDH9*
rs4701513	5:25925888	T/A	0.268	−0.11	−0.15, −0.07	5.44 × 10^−8^	intergenic	*LINC02211; CDH9*
rs10440666	5:25918597	G/A	0.267	−0.11	−0.15, −0.07	6.47 × 10^−8^	intergenic	*LINC02211; CDH9*
rs12697671	5:25929723	A/C	0.268	−0.11	−0.15, −0.07	6.61 × 10^−8^	intergenic	*LINC02211; CDH9*
rs72748996	5:25924670	G/A	0.268	−0.11	−0.15, −0.07	7.83 × 10^−8^	intergenic	*LINC02211; CDH9*
rs13164498	5:25931554	T/A	0.275	−0.11	−0.15, −0.07	7.98 × 10^−8^	intergenic	*LINC02211; CDH9*
rs6884731	5:25921084	G/A	0.268	−0.11	−0.15, −0.07	8.72 × 10^−8^	intergenic	*LINC02211; CDH9*
rs10440667	5:25919034	G/A	0.267	−0.11	−0.15, −0.07	9.58 × 10^−8^	intergenic	*LINC02211; CDH9*

^1^ Abbreviations: rsID—reference snp cluster id; CHR—chromosome number; BP—base position from hg38 build; A1—effect allele; A2—allele 2; EAF—effect allele frequency; CI—confidence interval; SBP—systolic blood pressure; DBP—diastolic blood pressure. ^2^ Associations are presented as standardized coefficients from additive genetic linear models. ^3^ Genome-wide significance *p* < 5.00 × 10^−8^ (**bold**).

**Table 4:** Top variants associated with systolic blood pressure response to chlorthalidone use over six months in treatment-naïve participants (*p* < 1.00 × 10^−7^) ^1^.

rsID	CHR:BP	A1/A2	EAF	β ^2^	95% CI	*p* ^3^	Location	Gene
**rs114758661**	**12:2472136**	**G/A**	**0.017**	**1.26**	**0.84, 1.68**	**1.12 × 10^−8^**	**intronic**	***CACNA1C***
**rs540857940**	**12:2475156**	**A/G**	**0.017**	**1.26**	**0.84, 1.68**	**1.12 × 10^−8^**	**intronic**	***CACNA1C***
**rs141391468**	**12:2470148**	**G/A**	**0.019**	**1.17**	**0.77, 1.57**	**3.59 × 10^−8^**	**intronic**	***CACNA1C***
**rs7905470**	**10:7247513**	**A/C**	**0.199**	**−0.44**	**−0.59, −0.29**	**4.72 × 10^−8^**	**intronic**	***SFMBT2***
rs80214621	5:157232090	T/G	0.017	1.40	0.91, 1.89	6.14 × 10^−8^	intronic	*ITK*

^1^ Abbreviations: rsID—reference snp cluster id; CHR—chromosome number; BP—base position from hg38 build; A1—effect allele; A2—allele 2; EAF—effect allele frequency; CI—confidence interval. ^2^ Associations are presented as standardized coefficients from additive genetic linear models. ^3^ Genome-wide significance *p* < 5.00 × 10^−8^ (**bold**).

## Data Availability

The raw GenHAT genotypic and phenotypic data used in this study are deposited in the National Center for Biotechnology Information (NCBI) Database for Genotypes and Phenotypes (dbGaP), accession number phs002716.v1.p1. The data for the Pharmacogenomic Evaluation of Antihypertensive Responses (PEAR) study and PEAR-2 have been made publicly available in dbGaP under accession: phs000649.v2.p2.

## Supplementary Materials

The following supporting information can be downloaded at: https://www.mdpi.com/article/10.3390/genes13071260/s1, Figure S1: Manhattan and QQ plot for the FG analysis results; Figure S2: QQ plots for the discovery BP response analysis; Figure S3: Marginal mean blood pressure response by genotype; Figure S4: LocusZoom regional plot for rs10440665 in the intergenic region on chromosome 5 between *LINC02211* and *CDH9*; Figure S5: LocusZoom regional plot for rs114758661 in the intronic region of *CACNA1C* on chromosome 12; Figure S6: LocusZoom regional plot for rs79772702 in the *GIMAP1-GIMAP5* readthrough region on chromosome 7. Table S1: Demographics of participants included in ICAPS validation; Table S2: Baseline Demographics of GenHAT Treatment-Naive Participants for ΔBP Analysis; Table S3: Chlorthalidone Blood Pressure and Glucose Response in Treatment-Naive Participants; Table S4: GenHAT discovery analyses genomic control (λ) values; Table S5: Top variants (*p* < 1.00 × 10^−6^) from the GenHAT ΔSBP response; Table S6: Top variants (*p* < 1.00 × 10^−6^) from the GenHAT ΔDBP response; Table S7: Top variants (*p* < 1.00 × 10^−6^) from the GenHAT ΔSBP response in 290 treatment-naive participants; Table S8: Top variants (*p* < 1.00 × 10^−6^) from the GenHAT DBP response in 290 treatment-naive participants; Table S9: Top variants (*p* < 1.00 × 10^−6^) from the GenHAT ΔFG response; Table S10: Previously published (since 2010) variants associated with BP response to thiazide diuretics mono- or combination-therapy; Table S11: Previously published (since 2010) antihypertensive pharmacogenomic studies associated with glycemic traits [76].
