# Supplementary material for: Genetic Contributors of Efficacy and Adverse Metabolic Effects of Chlorthalidone in African Americans from the Genetics of Hypertension Associated Treatments (GenHAT) Study
Source: Genes (Basel). 2022 Jul 15;13(7):1260. doi: 10.3390/genes13071260 (PMC9319619; doi:10.3390/genes13071260)
Supplement: Supplementary file 1 [file genes-13-01260-s001.zip › Supplementary Material_Final.pdf]

Supplementary Methods

Study participants and inclusion criteria

GERA (Genetic Epidemiology of Responses to Antihypertensives): GERA was a prospective interventional trial of hydrochlorothiazide (HCTZ) that enrolled approximately 300 EA and 300 AA participants who had uncomplicated stage 1-2 HTN (1). Participants were instructed to discontinue any previous antihypertensive medications for at least 4 weeks. The study drug, HCTZ 25 mg daily for 4 weeks or candesartan 16 mg daily for 2 weeks followed by 32 mg daily for 4 additional weeks, was administered orally once BP was elevated (office DBP ≥90 mmHg) (2). GERA participants were genotyped using the GeneChip Human Mapping 500k Array (Affymetrix, Santa Clara, CA, USA) using standard procedures. Genotyped variants were imputed to the TOPMed freeze 5 reference panel using minimac4 software. In the current study, 283 AA participants from GERA were utilized in replication efforts.

PEAR (Pharmacogenomic Evaluation of Antihypertensive Responses): PEAR was a multicenter, randomized clinical trial that aimed to evaluate the genetic variability of BP response of 768 study participants on HCTZ and/or the beta-blocker atenolol monotherapy for 9 weeks (3). After a mean washout period of 28 days, participants were randomized to receive either HCTZ 12.5 mg daily or atenolol 50 mg daily for 3 weeks, followed by dose titration to 25 mg and 100 mg daily respectively, for SBP > 120 mmHg and DBP > 70 mmHg. After 9 weeks, response to monotherapy was assessed (4). In the current study, the Human Omni1M Quad GWAS chip (Illumina Inc, San Diego, CA, USA) was used for genotyping and imputation was completed using the TOPMed Freeze 5 reference panel and minimac4 software. A total of 148 AA participants were randomized to HCTZ and were utilized in replication efforts.

PEAR2 (Pharmacogenomic Evaluation of Antihypertensive Responses 2): PEAR2 included a similar HTN population as PEAR but tested 100 mg daily metoprolol (beta-blocker) and 25 mg daily chlorthalidone as sequential monotherapy, with at least a 4-week washout period prior to each treatment (3). After 9 weeks, response to monotherapy was assessed (5). PEAR2 used the Human Omni2.5 GWAS chip with parallel genotyping and imputation methods. In the current study, 139 AA participants (chlorthalidone) from PEAR2 were utilized in replication efforts.

Supplementary Figures

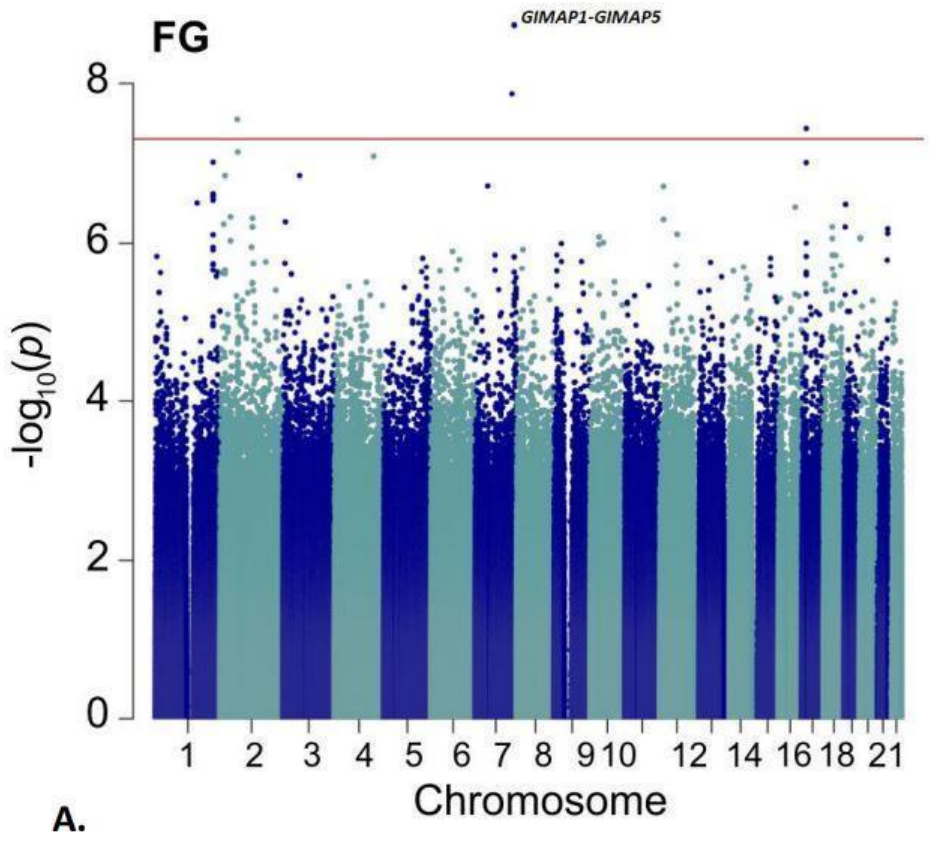

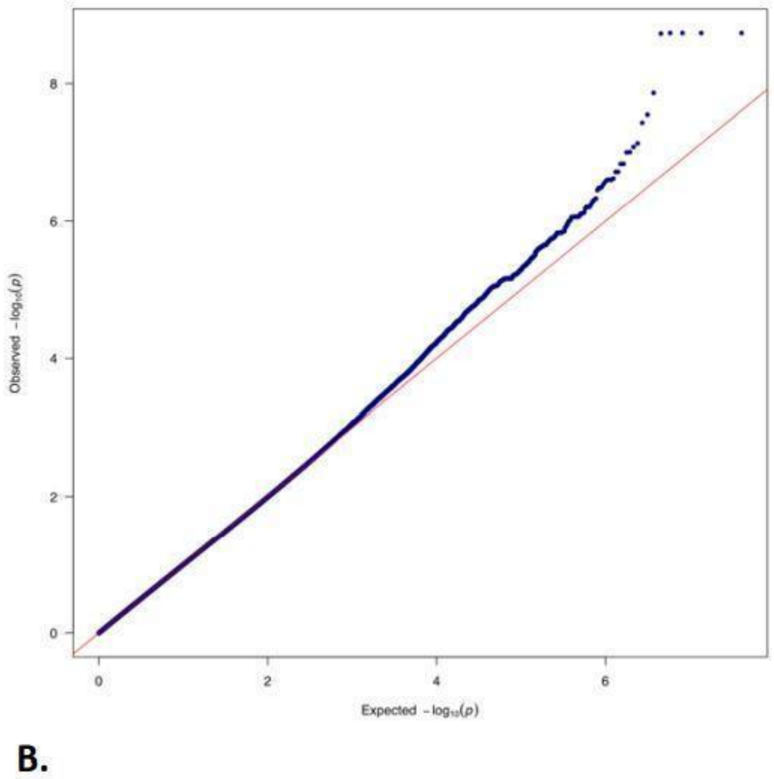

Figure S1. Manhattan and QQ plot for the FG analysis results. A; B) QQ plot for ΔFG analysis, lambda=1.012.

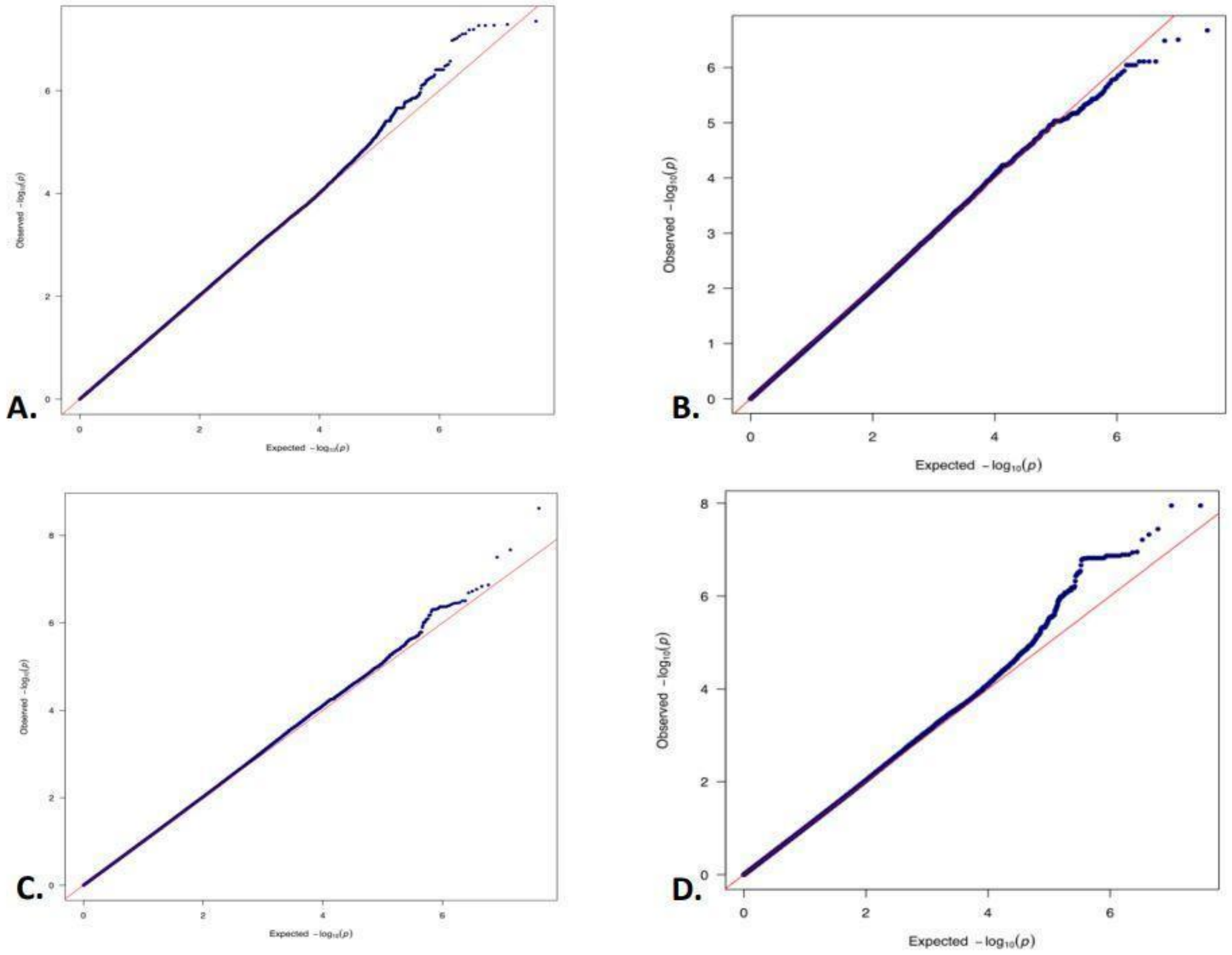

Figure S2. QQ plots for the discovery BP response analysis. A) ΔSBP analysis, lambda=1.008; B) ΔDBP analysis, lambda=1.005; C) ΔSBP treatment naive analysis, lambda=1.006; D) ΔDBP treatment naive analysis, lambda=0.985.

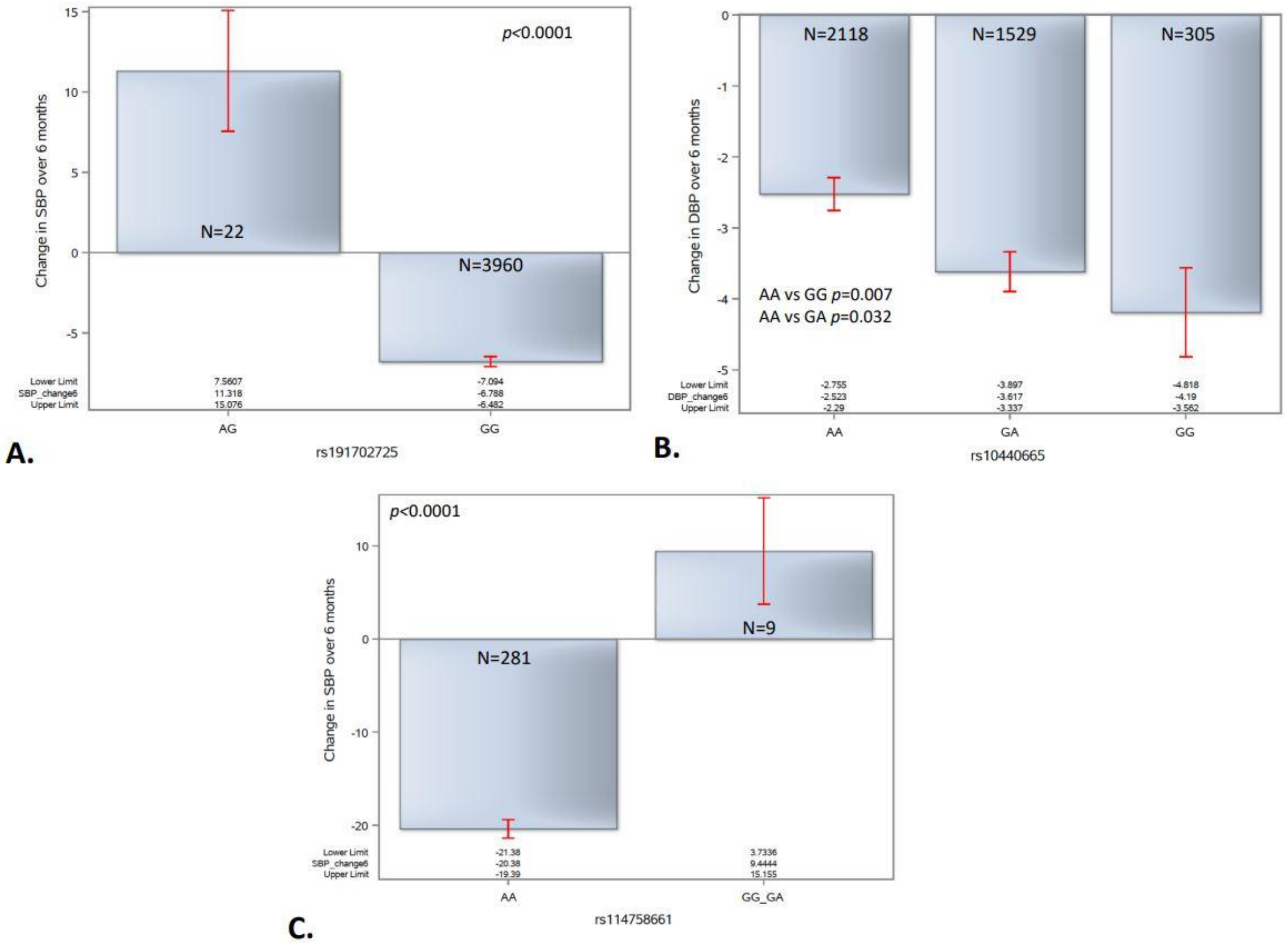

Figure S3. Marginal mean blood pressure response by genotype. A.) SBP response by genotype of rs191702725. B) DBP response by genotype of rs10440665. C.) SBP response in treatment naïve participants by genotype of rs114758661. Red error bars represent one standard error of the mean.

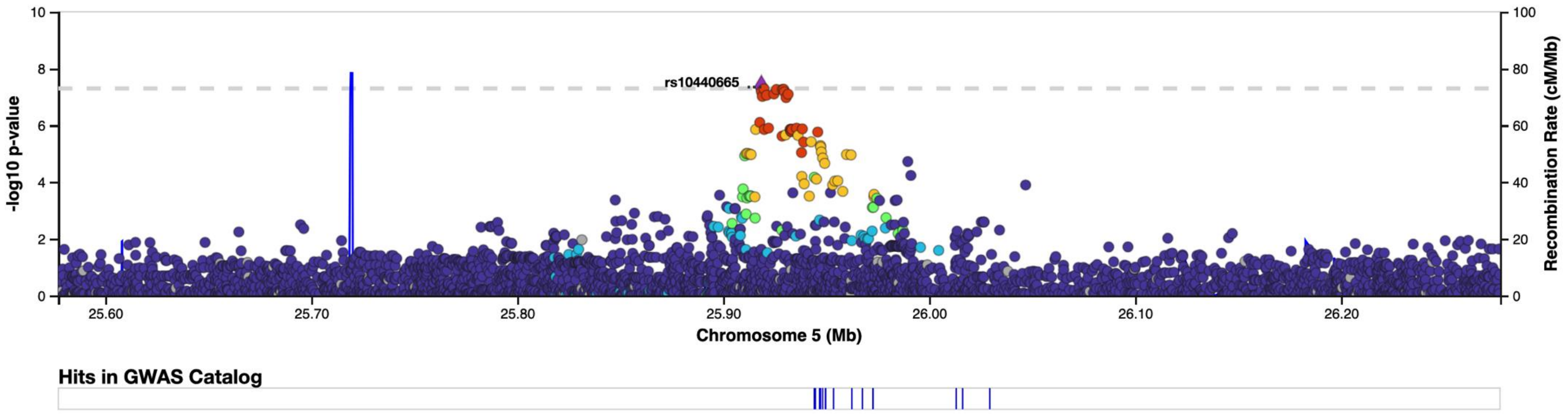

Figure S4. LocusZoom regional plot for rs10440665 in the intergenic region on chromosome 5 between *LINC02211* and *CDH9*. Annotation based on hg38 build.

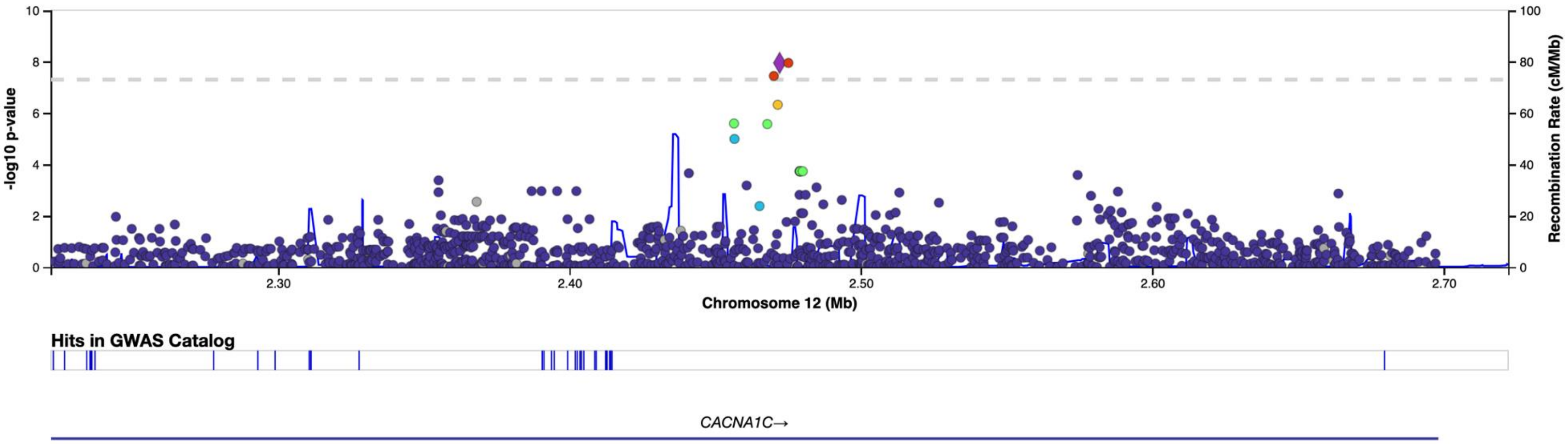

Figure S5. LocusZoom regional plot for rs114758661 in the intronic region of *CACNA1C* on chromosome 12. Annotation based on hg38 build.

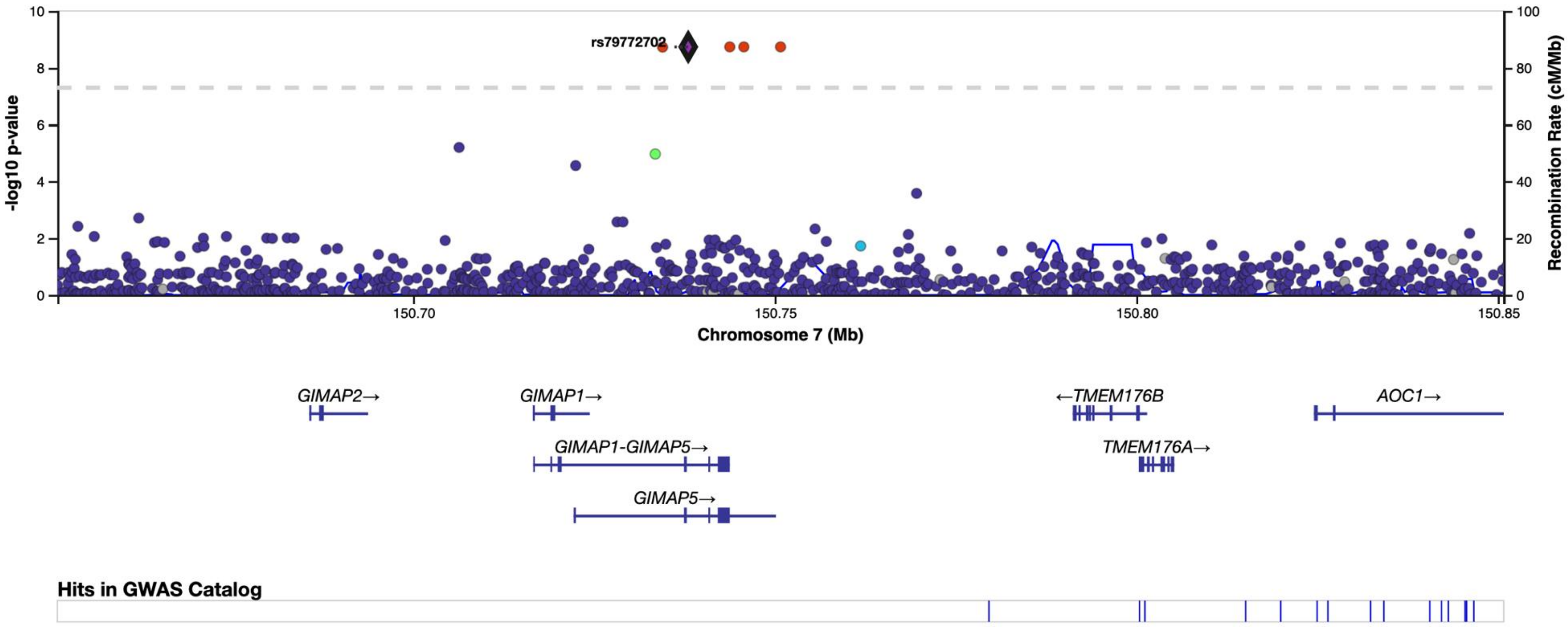

Figure S6. LocusZoom regional plot for rs79772702 in the *GIMAP1-GIMAP5* readthrough region on chromosome 7.
Annotation based on hg38 build

Supplementary References

1. Chapman AB, Schwartz GL, Boerwinkle E, Turner ST. Predictors of antihypertensive response to a standard dose of hydrochlorothiazide for essential hypertension. Kidney Int. 2002;61(3):1047-55.
2. Hiltunen TP, Donner KM, Sarin AP, Saarela J, Ripatti S, Chapman AB, et al. Pharmacogenomics of hypertension: a genome-wide, placebo-controlled cross-over study, using four classes of antihypertensive drugs. J Am Heart Assoc. 2015;4(1):e001521.
3. Sa ACC, Webb A, Gong Y, McDonough CW, Shahin MH, Datta S, et al. Blood pressure signature genes and blood pressure response to thiazide diuretics: results from the PEAR and PEAR-2 studies. BMC Med Genomics. 2018;11(1):55.
4. Johnson JA, Boerwinkle E, Zineh I, Chapman AB, Bailey K, Cooper-DeHoff RM, Gums J, Curry RW, Gong Y, Beitelshees AL, Schwartz G and Turner ST. Pharmacogenomics of antihypertensive drugs: rationale and design of the Pharmacogenomic Evaluation of Antihypertensive Responses (PEAR) study. Am Heart J. 2009;157:442-9. PMC Pmc2671287.
5. Hamadeh IS, Langaee TY, Dwivedi R, Garcia S, Burkley BM, Skaar TC, Chapman AB, Gums JG, Turner ST, Gong Y, Cooper-DeHoff RM and Johnson JA. Impact of CYP2D6 polymorphisms on clinical efficacy and tolerability of metoprolol tartrate. Clin Pharmacol Ther. 2014;96:175-81. PMC Pmc4111800
